# Survey of Applicable Methods for Determining Viscoelastic Effects in Ferroelectric and Antiferroelectric Chiral Liquid Crystals

**DOI:** 10.3390/ma17163993

**Published:** 2024-08-11

**Authors:** Dorota Dardas

**Affiliations:** Institute of Molecular Physics, Polish Academy of Sciences, 60-179 Poznań, Poland; dorota.dardas@ifmpan.poznan.pl

**Keywords:** viscoelasticity, ferroelectric liquid crystal, antiferroelectric liquid crystal

## Abstract

Viscosity, elasticity, and viscoelastic properties are one of the most fundamental properties of liquid crystalline materials; the main problem in determining these properties is the multitude of physical parameters needed to determine the values of elasticity and viscosity constants. In this paper, a number of different measurement methods for the complete characterization of viscoelastic properties for smectic liquid crystalline materials and their mixtures are analyzed, both theoretically and experimentally. The way in which viscoelastic material constants are determined depends mainly on the application/purpose of the materials under study. The subject of this work was to review the methods used to determine viscoelastic effects in ferroelectric and antiferroelectric chiral liquid crystals, their mixtures, composite materials, and even in dielectric systems, which would bear the hallmark of a universal method allowing the application of sufficiently low electric fields. In the case of chiral liquid crystals with ferroelectric and antiferroelectric phases and their subphases, the following assumption applies: fulfilment of Hooke’s law (in the case of elastic coefficients) and preservation of laminar flow (in the case of viscosity coefficients).

## 1. Introduction

Chiral liquid crystals [1,2,3] with ferroelectric and antiferroelectric properties have found numerous applications, mainly in optoelectronic devices such as light switches, modulators, displays, and video screens [4,5,6]. The use of chiral smectics makes it possible to improve greyscale, switching speed, contrast, information content, and other parameters of information imaging devices. Dielectric methods are mainly used in the study of ferroelectric and antiferroelectric properties [7,8], while electro-optical methods have mostly been used to obtain qualitative information [9,10]. It has been noted that the amplitude and phase of the electro-optical response can be additional parameters to help identify sub-phases of liquid crystals, as demonstrated in previous work [11,12]. This extended the possibility of using light modulation depth measurements to verify phase transition temperatures determined by other methods. It was important to show that the intensity of the second harmonic of the electro-optic response is a sensitive, useful indicator of the transition to the paraelectric smectic phase. A method was developed to measure the incident light intensity and the phase shift between the ordinary and extraordinary beam.

Generalized Debye equations have been proposed to describe the relaxation processes recorded by the electro-optical method. It is noted that some components have negative amplitudes [13]. Such a situation has no analogue in dielectric experiments. It was found that the modulation at the fundamental frequency is determined by a linear electro-optic coefficient, while the second harmonic by both coefficients: linear and quadratic. A method was then proposed to make the separation of the first and second harmonics possible. Additionally, a method for determining the electro-optic coefficients was proposed. This provided the possibility to determine the linear a and quadratic c electro-optic coefficients as a function of temperature for ferroelectric and antiferroelectric chiral smectic materials.

An important aspect in the dielectric and electro-optical studies of chiral liquid crystals was the demonstration of their complementarity [13] and the application of linear and nonlinear electro-optical effects [14,15] to the determination of the flexo- and piezoelectric polarization of smectic layers in ferroelectric and antiferroelectric liquid crystals [16]. In the course of scientific activities, due to the application-oriented nature of liquid crystal materials, it has become possible and important to pay particular attention to correct and authoritative methods for the determination of material properties of liquid crystals (not only nematic).

Viscoelastic effects are particularly important and are increasingly and interchangeably referred to as viscoelastic in materials engineering nomenclature [3,17,18,19,20].

Four parameters are of the greatest importance in information visualization devices and light modulators from the applications point of view: two elastic coefficients (for the torsional deformations of the smectic director c and the nematic director n) and two rotational viscosity coefficients for these directors [19]. Knowledge of the electro-optic coefficients has extended the research area to include the possibility of determining material parameters such as elasticity or viscosity in application-relevant mesogens. Subsequently, the work on the mechanical properties of chiral liquid crystals was clearly extended in the course of further research, which was directly related to the determination of elasticity and viscosity coefficients in chiral liquid crystals, with a particular emphasis on subphases with ferroelectric and antiferroelectric properties. The study of ferroelectrics was used to test the method and find the relationship between molecular structure and viscoelastic properties, which are important in the design of materials with desired properties and in the composition of mixtures of technical interest. The research of the viscoelastic properties of ferroelectric and antiferroelectric liquid crystals under small deformation conditions is a pioneering study of this kind. The results obtained provide a better understanding of the switching mechanisms in ferroelectric and antiferroelectric liquid crystals. Dielectric and optical methods have been used for deformation detection. Finding a relationship between dielectric and electro-optical measurements extended the capabilities of both methods. Due to the high sensitivity of the electro-optical method, it was possible to apply an extremely low electric field and make measurements in planar and homeotropically oriented samples. Tests on homeotropic samples provided data on the properties of volumetric samples, i.e., data free of surface-induced errors. Rotational viscosity coefficients were determined from dynamic effects (Goldstone and soft modulus relaxation). Detection of relaxation moduli was performed by dielectric and electro-optical methods.

## 2. Timeline of Measurement Concepts

### 2.1. Motivation and Aim of the Study

When talking about mechanical properties, one usually thinks of elastic properties in the case of solids and viscous properties in the case of liquids. Liquid crystals combine the properties of both solids and isotropic liquids. This combination of the elastic properties of solids (related to deformation) and the viscous properties of liquids (related to flow) creates some complications in the description of the mechanical properties of liquid crystals, which are therefore called viscoelastic properties. This combination creates a number of interesting phenomena not found in either solids or liquids [21]. In the case of ferroelectric liquid crystals, viscoelastic properties are important from a practical point of view, as they determine the switching rate and threshold voltage in displays. Among the group of liquid crystals whose mechanical properties are well understood, both theoretically and experimentally, we can include nematics [22,23,24]. Three independent elastic constants and five viscosity coefficients are needed for the mechanical properties of nematic liquid crystals. The above task is much more complicated in the case of smectic liquid crystals [10,25,26,27,28]. Here, a much larger number of parameters are needed due to the layered structure (and often lower symmetry). In the most extensive (but still simplified) model of chiral tilted smectics, developed by Dahl and Lagerwall [29], 15 elasticity constants describing only soft, volumetric deformations are included. In these materials, nine independent viscosity coefficients have to be considered, still without taking into account the deformation of the layers [30]. For this reason, the description of the mechanical properties of ferroelectric liquid crystals is very complex, even in its most simplified form. Therefore, some elasticity and viscosity coefficients have not yet been determined experimentally. The only available experimental work concerns the torsional elasticity and rotational viscosity of smectic c- and n-directors. These are coefficients of great technical interest.

### 2.2. Experimental Determination of Viscosity and Elasticity Coefficients

In isotropic fluids, the viscosity coefficient links the stress to the velocity gradients caused by the flow [31,32]. Similarly, the rotational viscosity coefficient γ links the torque Γ to the angular velocity of the particles:

(1) $$\Gamma =\gamma_{\varphi }\frac{d\varphi }{dt}$$

where φ is the angle of rotation of the molecules.

In the crystalline phase, elasticity coefficients link stresses to deformations. Similarly, in liquid crystals, the elasticity constants are the proportionality coefficients between torque stress and strain. For torsional deformation, we have:

(2) $$\Gamma =-K_{\varphi }\frac{d^{2}\varphi }{dz^{2}}$$

The coefficients of rotational viscosity γ and torsional elasticity *K_φ_* defined in Equations (1) and (2) are related to the Goldstone mod [27,28,29], i.e., to changes in the position of the inclination plane of the molecules without affecting the value of the inclination angle. To determine the viscosity coefficient, it is necessary to apply an external force to induce a controlled flow in the fluid [33,34]. Similarly, to determine the elastic coefficient, a controlled deformation should be induced by an electric field. In ferroelectric liquid crystals, an electric field can be used as the source of such an external force. Thanks to its coupling to the spontaneous polarization of smectic layers, flow or deformation can be easily induced [35,36,37]. In order to develop an experimental method to determine the above coefficients, the two following problems need to be solved:

(1) The value of the angle φ has to be determined from measurements of macroscopic quantities (such as refractive indices or electrical permittivity);
(2) The equation of motion in the c-direction has to be solved, i.e., a well-defined distribution of the azimuthal angle φ on a microscopic scale.

The equation of motion of the c-director has the form [21,38]:

(3) $$-K_{\varphi }\frac{\delta^{2}\varphi }{\delta z^{2}}+\gamma_{\varphi }\frac{\delta \varphi }{\delta t}=M$$

where M is the torque acting per unit volume of the liquid crystal. If the external torque is due to an electric field E, then:

(4) M=P×E

where P denotes spontaneous polarization.

The solution of the equation of motion (Equation (3)) depends on the shape and amplitude of the applied electric field. The procedure used to determine the γ and $K_{\varphi }$ coefficients depends on the method by which the deformation of the director c is detected. The methods used to determine the rotational viscosity coefficient γ are summarized in Table 1. Table 2 collects the various methods used to determine the torsional elasticity constant of the c-director.

The number of experimental papers devoted to the measurement of viscosity and elasticity constants in tilted smectic phases is relatively small [40,41,42,43,44,45,46,47,48]. The methods listed in Table 1 and Table 2 have been used to test different materials. However, there are no results obtained for the same material by different methods. In this case, it is practically impossible to compare the methods and their results and their variation can be explained by the material properties.

For the elastic constant $K_{\varphi }$, the experimental results differ by two orders of magnitude [44,46]. The main reason for these discrepancies is probably due to nonlinear effects. The definitions of viscosity and elasticity coefficients assume linear dependence of angular velocity and strain on torque (see Equations (1) and (2)). This is only true for laminar flows and small deformations. Therefore, if large deformations or turbulent flows occur during the measurement, this can cause significant systematic errors. By extension, all methods in which a high electric field is applied, such as the switching method [39,40,41,42] or the critical field method [46], have to be used with extreme caution. Low-strain methods [43,44,47,48] seem to be more reliable and can be considered as standard methods.

The study of liquid crystals and their applications has seen significant progress in recent years [49,50]. Traditionally, researchers have focused on applications in liquid crystal displays and optical shutter elements. Nowadays, the spectrum of possibilities includes entirely new and diverse applications in the field of nanotechnology and nanoscience Stabilization of the mesophase is currently being explored. Furthermore, extending and controlling its temperature range through the use of polymers and biopolymers [51] is particularly relevant for phases characterized by a very narrow temperature range, a 0.12 °C range, for example, in so-called “exotic liquid phases”. Examples of exotic liquid crystalline phases are the blue phases (BPs), which are characterized by the dispersion of a Kerr constant [52] or in chiral smectic phases (SmC*_α_, SmC*_β_, and SmC*_γ_) [53]. Functional nanoparticles are also being used to modify the physical properties of liquid crystals by adding ferroelectric [54], antiferroelectric [55], or magnetic particles in a wide variety of shapes and sizes. Hybrid materials, where the liquid crystal is doped with particles that affect optical properties, such as quantum dots, plasmonic, semiconductor, or metamaterials, are also of increasing interest. Self-organization of liquid crystals is used to order and orient matrices, which is currently used in smart glasses or liquid crystal elastomers, among other applications [56].

In most applications of liquid crystal materials, it is important, and sometimes essential, to know a large number of parameters (including elasticity and viscosity coefficients) that influence the behavior of the material under study under controlled and defined conditions [57,58,59,60]. The lack of a reference point in relation to chiral liquid crystalline materials, in particular those with ferroelectric and antiferroelectric properties, was my primary motivation to start my research on the determination of the elastic coefficient and rotational viscosity. Knowledge of the material properties of liquid crystals and their influence on the structure, ordering, and characteristics of a given mesophase and neighboring phases is essential for the design of high-performance optoelectronic components such as liquid crystal displays, light modulators, or filters. As mentioned earlier, it is relatively common to determine elasticity and viscosity constants in a strong electric field (which often induces an overshoot of the proportionality range of the elasticity constant and generates turbulent flow, while it should remain laminar); weak electric fields are rarely used, although they are important in assessing the applicability of a material. The type and nature of the phase transformations occurring in the material under investigation can have a decisive influence not only on the value of the determined coefficients but also on their temperature dependence.

The aim of this study was to review the methods used to determine viscoelastic effects in ferroelectric and antiferroelectric chiral liquid crystals, their mixtures, composite materials, and even in dielectric systems that would have the hallmarks of a universal method, allowing the use of sufficiently low electric fields. In the case of chiral liquid crystals with ferroelectric phases, antiferroelectric phases, and their subphases, the following assumption applies: satisfying Hooke’s law (in the case of elastic coefficients) and preserving laminar flow (in the case of viscosity coefficients).

### 2.3. Theoretical Background

To measure any coefficient of elasticity, one must apply a suitable stress to a suitably oriented specimen and then measure the strain caused by that stress. Both stress and strain should be small enough to satisfy the proportionality condition between strain and stress (Hook’s law).

For chiral C* smectics, this means that the change in the angle between the c-directions in adjacent smectic layers has to be small compared to the equilibrium value of this angle. Due to the ferroelectric polarizations of the smectic layers in the SmC* phase, the simplest way to introduce deformation is to apply an electric field parallel to the smectic layers. In this case, the low deformation condition means that the electric field strength is much lower than the critical field EC needed for helix unfolding.

Studies of the mechanical properties of the c-director in ferroelectric smectics with an undeformed or weakly deformed helical structure [30] are still scarce. An electro-optical method has been proposed for the determination of the rotational viscosity coefficient γ [39] and the torsional elasticity coefficient B3 [38,61] (according to the notation [29], later papers used the designation $K_{\varphi }$ and, finally, K) for a smectic c-director, i.e., the projection of a nematic n-director onto the plane of the layer [21]. In the following description, I will consistently use the designation $K_{\varphi }$ for the elasticity coefficient and γ for the rotational viscosity coefficient. This method satisfies the conditions of low deformation and laminar flow. Furthermore, it was decided to carry out the measurement in homeotropically oriented samples, in which the helix is much less deformed than in the usual planar samples. In the papers [62,63], it was decided to limit ourselves to the determination of the rotational viscosity coefficient γ and the torsional elasticity constant $K_{\varphi }$. These coefficients strongly affect the properties of ferroelectric liquid-crystal-based displays. A separate problem is the deformation detection method. Deformation of the molecular distribution causes a change in the macroscopic properties of the sample. As optical properties are relatively easy to detect, the electro-optical method was therefore used to determine deformation. For the numerical estimation of $K_{\varphi }$ and γ, it is necessary to assume models linking both the deformation of the molecular distribution to the strength of the electric field and the molecular distribution to the macroscopic optical properties of the sample. In the ideal undisturbed chiral C* smectic layer, the position of the c-director in successive smectic layers is described by:

(5) $$\varphi =\frac{2\pi \cdot z}{p}$$

where φ is the azimuthal angle between the c-direction and an arbitrary x axis of the Cartesian co-ordinate system, *z* is the co-ordinate in the direction normal to the smectic layers, while *p* is the helix pitch. When an electric field is applied parallel to the smectic layers, the effective dipole moment of the smectic layers tends to reorient towards the electric field. This reorientation is hindered by elastic and viscous forces. Since the properties of the c-diode are similar to those of the n-diode nematic, we can use the nematodynamic equation to describe the ϕ azimuthal angle [30,61]:

(6) $$K_{\varphi }\frac{\partial^{2}\varphi }{\partial z^{2}}-\gamma \frac{\partial \varphi }{\partial t}=P_{S}\cdot E\sin\varphi$$

where $P_{S}$ is the spontaneous polarization and $E=E_{0}\cos t$ denotes the electric field strength with an angular frequency of *ω* applied to the sample. For small deformations, the steady-state solution of Equation (5) is of the form:

(7) $$\varphi =qz+\varphi_{0}\cdot \cos\left(\omega t+\beta \right)\;\cdot \sin qz$$

where q=2 and the amplitude of the change in angle φ is $\varphi_{0}$:

(8) $$\varphi_{0}=\frac{P_{S}\cdot E_{0}}{\gamma }\cdot \frac{1}{\sqrt{\frac{1}{\tau^{2}}+\omega^{2}}}$$

and β=arctanωt, where *τ* is the relaxation time:

(9) $$\tau =\frac{\gamma }{K_{\varphi }\cdot q^{2}}$$

The second term of Equation (7) describes the response of the chiral smectic C* to the applied electric field AC. The angle changes are also periodic but shifted by an angle *β*. The deformation of the helix described by Equation (8) causes changes in the dielectric and optical properties of the chiral C* smectic. One easily detectable change is the shift in the optical axis. When the inclination angle of the $\varphi_{0}$ molecule is small, the relationship between the amplitude of the azimuthal angle and the amplitude of the inclination of the optical axis has the following approximate form [61]:

(10) $$\vartheta_{0}=\frac{1}{2}\varphi_{0}\cdot \theta$$

Thus, measuring the amplitude of the slope of the optical axis at different frequencies makes it possible to determine $K_{\vartheta 0}$ and γ using Equations (8) and (9). When γ << 1/*τ*, then ϑ0 does not depend on frequency and, from Equations (8)–(10), we obtain [62]:

(11) $$K_{\varphi }=\frac{P_{S}\cdot E_{0}\cdot \theta }{2\vartheta_{0}\cdot q^{2}}$$

From the frequency dependence of the ϑ0, the relaxation time of the τ can be calculated, followed by the viscosity of the γ [63]:

(12) $$\gamma =K_{\varphi }\cdot q^{2}\cdot \tau =\frac{P_{S}\cdot E_{0}\cdot \theta }{2\vartheta_{0}}\tau$$

where τ—relaxation time, $\tau_{s}$—switching time, A—electrode area, $K_{\varphi }$—torsional elasticity constant for the c-director, E—electric field, i—light intensity, $P\;,P_{S}—$spontaneous polarisation, $q=\frac{2\pi }{p}-wave\;factor\;of\;helix$, p—pitch, and $\vartheta_{0}$—azimuth angle.

As shown earlier, the field-induced collective motion of molecules in smectic ferroelectric liquid crystals with helical superstructure is usually studied by analyzing the fluctuation of the azimuthal angle ϑ along the *z* axis normal to the smectic layers. In addition, it is assumed that the molecules do not interpenetrate each other and the smectic layers are well defined. Then, the rotational motion of the molecules induced by an external electric field varying sinusoidally with amplitude $E_{0}$ and angular frequency ω is described by Equation (6) [1,9].

In the case of antiferroelectric liquid crystals, the polarization of each pair of adjacent smectic layers is almost abolished but, due to the presence of a helical structure, there is still a nonzero value [1]. Since the thickness of the smectic layer l is much smaller than the helix pitch p, the absolute value of the remaining local polarization associated with a pair of smectic layers can be determined by the relation:

(13) $$\delta P_{S}=2\pi \frac{l}{p}P_{S}$$

Given the solution of the equation of motion of a pair of molecules belonging to adjacent smectic layers, it can easily be shown, by analogy with Equation (11), that, for antiferroelectric liquid crystals, the solution can be obtained simply by replacing $P_{S}$ in Equation (6) by $\delta P_{S}$ [64]. Then, using Equations (10), (11) and (13) and considering that $l=l_{0}cos\theta$, where ${\;l}_{0}$ is the length of the molecule (the linear size of the molecule measured in the direction parallel to the n-director), we obtain:

(14) $$K_{a}=\frac{P_{S}\cdot E_{0}\cdot l_{0}\cdot sin2\theta }{4\vartheta_{0}\cdot q^{2}}$$

The right side of the above relationship includes parameters that can be determined experimentally. To calculate $\vartheta_{0}$, the method of measuring the linear depth of light [29] was used, which will be presented in more detail when describing the experiment. Thus, this equation allows us to calculate the torsional elasticity constant for an antiferroelectric liquid crystal in a weak electric field [65,66].

The change in the orientation of the optical axis ∆α in a weak electric field can be expressed as [2]:

(15) $$∆\alpha =aE_{0}$$

with a coefficient a independent of *E*_0_. The linear electro-optical coefficient a [12] describing the change in orientation of the optical axis under the influence of a weak electric field *E* that does not destroy the helical structure can be expressed as:

(16) $$a=\frac{1}{2}\frac{P_{S}\sin\theta }{Kq^{2}\sqrt{1+\omega^{2}\tau^{2}}}$$

where q=2π/p, *p* is the helix pitch, *θ* the angle of inclination of the molecules in the smectic layer (in the ferroelectric smectic phase C*, chiral molecules are spontaneously inclined with respect to the axis normal to the layer), and *τ* denotes the relaxation time of the Goldstone modulus (9). Solving Equation (6) for small deformations, after taking into account Equation (16), for the condition *τ* << 1, the expression for the interlayer elasticity factor $K_{\varphi }$ [67,68,69] can be obtained in the form:

(17) $$K_{\varphi }=\frac{1}{8\pi^{2}}\frac{P_{S}\cdot p^{2}\cdot sin\theta }{a}$$

Equation (17) allows calculation of the coefficient $K_{\varphi }$ when the following parameters are known: a (linear electro-optical coefficient), $P_{0}\;$(local spontaneous polarisation), p (pitch), and θ (angle of inclination of molecules). In addition, from Equations (9) and (17), if τ is known, the rotational viscosity can be determined [70]:

(18) $$\gamma =\frac{P_{S}\cdot \theta }{2a}\tau$$

where τ is the relaxation frequency of the Goldstone modulus, that is, the relaxation mod associated with fluctuations of the long axis of the molecules in the azimuthal direction with respect to the normal to the smectic layer. The formulas in Equations (17) and (18) are the basis for determining the viscoelasticity in ferroelectric helical liquid crystals in the small deformation limit and the laminar flow limit. In the case of antiferroelectric liquid crystals, after considering Equation (13), we obtain [71]:

(19) $$K_{a}=\frac{1}{8\pi^{2}}\frac{{\delta P}_{S}\cdot p^{2}\cdot sin\theta }{a}$$

and:

(20) $$\gamma_{a}=\frac{{\delta P}_{S}\cdot \theta }{2a}\tau$$

The formulas in Equations (19) and (20) are the basis for determining the viscoelasticity in ferroelectric liquid crystals with helical structure in the limit of small deformations and the limit of laminar flow.

An expanded list of available methods for determining the viscoelasticity of $K_{\varphi }$ and $K_{a}$ in chiral smectics with ferroelectric and antiferroelectric properties is presented in Table 3, while an expanded list of available methods for determining the rotational viscosity of γ and $\gamma_{a}$ is presented in Table 4. $K_{\varphi }$ and $K_{a}$ denote the coefficients of elasticity in the ferroelectric and antiferroelectric phases, while γ and γ a denote the rotational viscosity in the ferroelectric and antiferroelectric phases, respectively.

## 3. Materials and Methods

The subjects of this study were smectic chiral liquid crystals, differing in the sequence of phase transitions. The first of these was 4′-methylbutylxy phenyl-4-acetylxy-benzoate material, designated as C8 [62,63,68]—showing only two mesophases. The ferroelectric phase appears in it immediately after softening; then, we observe a transition to the paraelectric phase of the twisted nematic. This material has been tested by various methods used to date [68]. Another commercially available selected material was 4-(n-hexylxy phenyl)-1-(2-fuethyl butyl) biphenyl-4-carboxylate, called Ce-3 [69,70], whose phase sequence was analogous to C8, and 4-(2-methylbutyl) phenyl-4-n-octylbiphenyl-4-carboxylate, Ce-8 [69,70], a ferroelectric liquid crystal, which, in turn, exhibits a very rich polymorphism; from the smectic phase C* during heating, we observe a phase transition to the non-spiral, paraelectric smectic phase A*, then to the chiral, nematic phase N*, and then to the blue phase, whose periodicity is formed by a superstructure of three-dimensional defects. In the group of materials that exhibit antiferroelectric properties is 4-(1-methyl-heptyloxycarbonyl) phenyl 4′-(3-butanoyloxy propyl-1-oxy) biphenyl-4-carboxylate, abbreviated as D12 [65,66], synthesized at the Military University of Technology in Warsaw, Poland (see Table 5). This material is characterized by a phase transition directly from the antiferroelectric phase SmC* to the paraelectric phase A*. The last material presented is 4-(1-methylheptyloxycarbonyl)phenyl 4-acetylxybiphenyl-4-carboxylate, a commercially available material (Military University of Technology, Warsaw, Poland, Sigma-Aldrich, Saint Louis, MO, USA) exhibiting rich polymesomorphism, with the acronym MHPOBC [71]. Phase transitions from the antiferroelectric SmC*_A_ and ferroelectric SmC* phases to the paraelectric SmA* phase take place through so-called exotic phases with ferroelectric properties, respectively, SmC*_γ_ and SmC*_α_.

A typical electro-optical measurement cell consists of two glass plates separated by spacers. This ensures that the inserted layer of liquid crystal has a well-defined thickness. For measurements with planar ordering, commercial cells manufactured by Instec (Instec Inc., Boulder, CO, USA) with a thickness of 5 µm, AWAT (Warsaw, Poland) with a thickness of 5 µm, and EHC (EHC Co., Ltd, Tokyo, Japan) with a thickness of 12 µm, 30 µm, and 110 µm were used. The conductive surfaces (electrodes) are made of indium tin oxide and coated with a polymer orientation layer. Thin electrical wires are attached to the electrodes. The cells used for measurements with homeotropic ordering were prepared using two strips of aluminum foil glued to a glass plate at a distance of about 1–1.6 mm. Another glass plate was attached to the top of the electrodes. Both glass plates were coated with a layer of surfactant (hexadecyl-trimethylammonium bromide) to achieve homeotropic boundary conditions. The temperature suitable for filling the cell is that of an isotropic liquid. A drop of liquid is placed near the gap between the slides, which, due to the capillary effect, fills the entire cell. We can use the cell prepared in this way for thermo-optical, electro-optical, dielectric, and phase transition observations. In order to obtain a homogeneous orientation of the smectic layers of the liquid crystal, perpendicular or parallel to the electrodes, various procedures are often used. Examples include very slow cooling from the isotropic phase, introducing a temperature gradient near the phase transition, using a suitably modulated voltage, or applying a DC voltage. There is no single universal method. Thin cells provided better ordering of smectic layers but strongly deformed the helix. On the other hand, thick samples did not significantly affect the structure of the helix but caused worse ordering of the layers. The ordering of the sample was checked by using a polarizing microscope.

In order to determine the rotational viscosity coefficient γ and the torsional elasticity constant $K_{\varphi }$ for chiral smectics with ferroelectric and antiferroelectric properties, the following quantities have to be measured:

- The spontaneous polarization of *P_S_*;
- The pitch *p*;
- Inclination angle *θ*;
- Linear electro-optical coefficient a;
- The relaxation time *τ*.

Analyzing the equations presented, it is not difficult to see that the value of elasticity and viscosity coefficients in liquid crystals with ferroelectric and antiferroelectric properties is influenced by spontaneous polarization, the tilt angle of the molecules, and, in particular, the helix pitch. Spontaneous polarization identifies the coupling of the applied electric field to the dipole moment of the molecules, the layers, and the volume response and the tilt angle of the molecules influences the electro-optical response of the system under study. However, it is worth emphasizing that it is the precise, conformal measurement of the helix pitch that can determine the validity of the results obtained. This is mainly due to the quadratic dependence of the wave vector of the helix for both the elastic coefficient and the rotational viscosity. For this reason, the determination of the helix pitch in smectics with properties should be carried out under volumetric conditions (bulk). In the case of chiral smectics, such conditions are met when the planar arrangement allows an undeformed helix to unfold, which requires a large cell thickness (and an applied electric field) appropriate to the pitch of the helix or a homeotropic arrangement that allows the helix to unfold unconstrained, parallel to the limiting surfaces.

- Spontaneous polarization was measured by a standard method, using a Diamant- Pepinsky bridge [67]. Spontaneous polarization [62,63,68] was determined in homeotropic cells, while, for [66,69,70,71], in planar cells.
- The pitch was measured in homeotropically oriented samples using the following methods: spectroscopic [62,68,69,70] and the Cano method in a wedge sample [62,68] and in a homeotropically oriented free liquid crystal droplet [71]. The aforementioned methods give comparable results under the assumption that the refractive index of the common ray is 1.4.
- The tilt angle of the molecules was measured using the alternating field method. An electric field of rectangular shape with a fairly high frequency (from tens to hundreds of hertz) was used here. Light intensity was measured using a photodiode and observed on an oscilloscope.
- Changing the position of the optical axis causes a change in the intensity of light passing through the sample. The intensity of the light is recorded by a photodiode. At the output of the preamplifier, a signal proportional to the light intensity is obtained. The signal modulated in this way contains, in addition to a constant component, a variable component, called the electro-optical response. In addition, changes in the angle α can be simulated by rotating the microscope table along with the sample by a small angle αK [65,66]. In the work [69,70,71], an additional modification of this method was used. In the experiment, the constant component of the intensity of light passing through the sample was measured simultaneously with the variable component. During the measurement of the electro-optical response, the sample made oscillations with an amplitude of 0, 14°. The frequency of the oscillations was determined by the speed of rotation of the synchronous motor putting the system into motion and was 6.25 Hz. Calibration of the electro-optical response using the above-described system made it possible to obtain values of the light modulation depth in absolute units.
- Relaxation time was determined from measurements of the real and imaginary components of the electro-optical response as a function of frequency.

The main principles of measuring viscoelastic effects using the electro-optical method and step-by-step linear measurement conditions are as follows: first, the material to be tested by the capillary method is placed in an electro-optical cell with a defined planar order and thickness. The sample prepared in this way is placed in a heating oven connected to a measuring system with the possibility of precise temperature control and dielectric, electro-optical measurements, and electro-optical response detection with the simultaneous possibility of observing the material with a polarizing microscope. This should be followed by a procedure for homogeneous ordering of the sample; the most effective method has proven to be very slow cooling from the isotropic phase in a slowly varying electric field. When the material is in a smectic, ferroelectric phase, we position it at 22.5 deg between crossed polarizers, where the electro-optical response shows a maximum. To determine the linear electro-optical coefficient a, the real and imaginary components of the electro-optical response should be measured as a function of temperature; to determine the relaxation time, it should also be measured as a function of frequency [12,66]. Both relationships are measured simultaneously using a lock-in amplifier during the oscillation of the microscope table caused by the stepping motor with the photodiode, allowing the electro-optical response to be calibrated and absolute values to be obtained. Then, without taking the sample out of the oven, with the same temperature controller, the spontaneous polarization $P_{S}\;$ in the Diamant system and the tilt angle θ of the molecules are measured using an oscilloscope. A separate experiment is required to determine the pitch in the volume cell (with homeotropic ordering, preferably in a free droplet). The measurement requires the use of a spectrophotometer. The results obtained in this way are used to determine the coefficient of elasticity and rotational viscosity.

## 4. Results and Discussion

**Figure 1:**
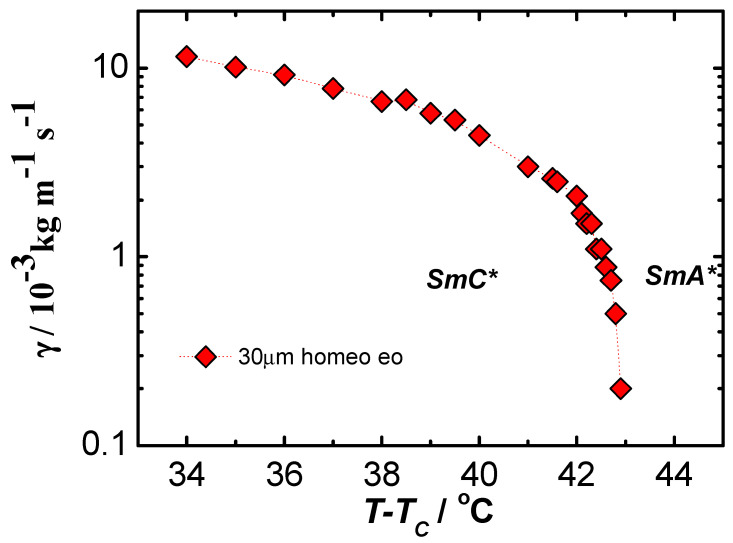
Temperature dependence of the rotational viscosity coefficient γ in a ferroelectric liquid crystal C8.

**Figure 2:**
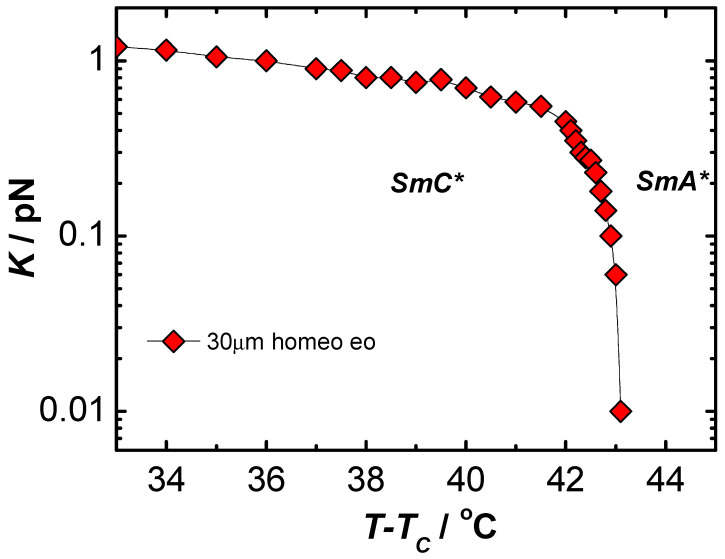
Temperature dependence of the elastic coefficient $K_{\varphi }\;$ in a ferroelectric liquid crystal C8.

On the basis of measurements made by complementary methods of many physical quantities, it was possible to determine the elastic coefficient $K_{\varphi }$ and rotational viscosity γ in liquid crystalline materials with ferroelectric properties [62,63]. It was assumed that the parameters corresponding mostly to the behavior of volume samples should have at least deformed helical structure. Disturbances of the helix of chiral smectics can be caused by too strong anchoring conditions in thin planar samples, as well as by the use of too strong electric fields that cause a complete pitch disruption and saturation state in thicker samples. The results obtained for the ferroelectric C8 material obtained under conditions similar to the bulk sample (homeotropic ordering) and in weak electric fields (guaranteeing the linearity and reversibility of the studied effects, according to Hooke’s law, and with a relatively low flow velocity of the liquid crystal guaranteeing its laminarity [62,63]) are presented in Figure 1 and Figure 2.

The rotational viscosity coefficient γ disappears at the SmC*–SmA* phase transition. This is not surprising, since this parameter describes the properties of the c-director, which disappears at the transition to a non-tilted phase that no longer has a helicoidal structure. Comparison of our experimental results with literature data is difficult, since data on material constants are very sparse. The scatter of measurement results is very large (more than two orders of magnitude). The literature data are mostly obtained for flat samples, and we suppose that the results are strongly dependent on the geometry of the sample. In addition, it is difficult to compare results for different materials. The values of the rotational viscosity coefficient obtained in this article agree fairly well with some literature data [21,33,72] but are clearly very different from many others obtained in planar orientation; see, for example, [73,74,75].

When analyzing the results, special attention should be paid to determining the pitch p to ensure correct values of the elastic constant $K_{\varphi }$. The pitch should be measured with high accuracy, since its square appears in Equation (7). The stresses present in the helix can affect the response of the optical axis of the sample due to the electric field [76]. The best way to avoid the influence of stresses on the determined values of the elastic constant is to measure the inclination of θ and the inclination of the optical axis $\vartheta_{0}$ at the same time or at least in the same sample geometry. Therefore, the results of measurements of the inclination of molecules in homeotropic samples were used to calculate $K_{\varphi }$. The elasticity constant $K_{\varphi }$ vanishes at the SmC*–SmA* phase transition. Comparison of our experimental results with literature data is difficult because, so far, measurements of the volume elasticity coefficient $K_{\varphi }$ in weak fields have been described only in two papers [76,77].

The $K_{\varphi }$ values obtained in these works are similar to those presented here (0.5 × 10^−12^ N, 1 × 10^−12^ N, and 0.9 × 10−12 N, respectively, at temperatures 3 × 10^−12^ N below T_CA_). However, data obtained for planar samples are much more scattered (for example, [72,73,75]). Most of these are based on dielectric measurements, which are strongly dependent on the sample geometry [26,44,61].

**Figure 3:**
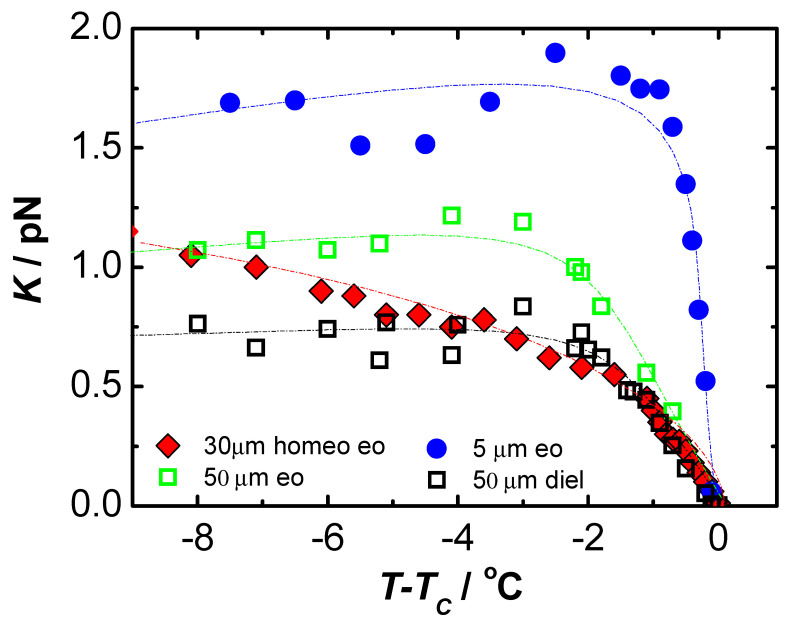
Temperature dependence of the elastic coefficient $K_{\varphi }$ in ferroelectric liquid crystal C8 obtained by various methods. In the diagram, ”eo” denotes electro-optical methods and “diel” dielectric methods. Thicknesses of planar samples, 5 µm and 50 µm; thickness of homeotropic sample, 30 µm.

**Figure 4:**
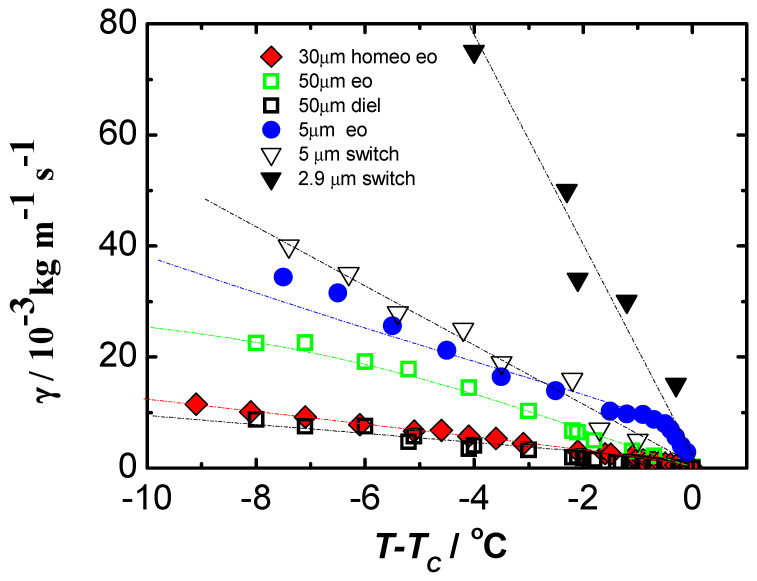
Temperature dependence of the rotational viscosity coefficient γ in ferroelectric liquid crystal C8 obtained by various methods. In the diagram, “eo” denotes electro-optic methods, “diel” dielectric, and “switch” switching methods. Thicknesses of the planar samples, 2.9 µm, 5 µm, and 50 µm; thickness of the homeotropic sample, 30 µm.

However, it is difficult to make any conclusive statements because, here too, the results apply to different materials. In summary, values of viscoelastic coefficients were obtained using a weak electric field. All measurements were made in cells with homeotropic ordering, which allows one to get significantly closer to the volume properties of the material under study. The results obtained look promising, so it was next decided to test the material 4′-methylbutylxy phenyl-4-octyloxy-benzoate, called C8, a liquid crystal with ferroelectric properties by available methods, and thus compare the results obtained for one material versus temperature. The results of such an analysis are shown in Figure 3 for the value of the obtained elastic coefficient $K_{\varphi }$ and in Figure 4 for the rotational viscosity γ.

Figure 3 clearly shows that the experimental conditions (primarily thickness and type of ordering) have a strong influence on the measurement results [68]. The results differ not only in values but also in the shape of the temperature dependence. It is worth noting that the temperature dependencies are rather irregular for planar-ordered samples. A similar behavior was observed for the rotational viscosity coefficient γ, shown in Figure 4. It was supposed that surface interactions are the main reason for the differences in the results obtained under different experimental conditions. The largest values of $K_{\varphi }$ coefficients were obtained for a thin (5 µm), planar-ordered sample, where surface interactions play the most important role.

Since the helical structure of the smectic SmC* phase is not always compatible with planar ordering, the properties of the cell greatly affect the measurement results. In the case of homeotropic orientation, the helical structure and other material properties have less influence on the measurement results and are less surface-dependent. The smectic layers of the homeotropically oriented sample are parallel to the cell walls, and the helical structure is only slightly deformed near the surface. We believe that the results obtained in thick homeotropic cells better reflect the bulk properties of the chiral smectic phase C*. Therefore, we assume that we can consider these results as reference results. As can be seen in Figure 3, there is some correlation between the dielectric results obtained for the 50 µm planar sample and the 30 µm homeotropic sample. The discrepancy may be due to the different temperature dependence in the planar and homeotropic sample [77]. The helix pitch should always be measured with high accuracy [76], since its square appears in theoretical formulas for viscoelasticity coefficients [62,64]. It is obvious that the stresses deforming the helix can affect the response of the optical axis to the applied electric field and consequently cause fluctuations in the value of the $K_{\varphi }$ parameter.

Figure 4 shows the results of rotational viscosity γ determined by various methods. Abbreviations were used, where “diel” stands for dielectric methods, “eo” electro-optical, “switch” switching method, and “homeo eo” our proposed method for testing a near- volumetric sample. As you can see, the discrepancy in results is significant. The switch method used to determine the rotational viscosity coefficient γ requires special attention. It is the most widely used method. As Figure 4 shows, the results obtained by this method differ significantly from the results of all other methods. Moreover, the dependence on cell thickness is also the greatest. Therefore, the results obtained by the switching method should be treated with great caution.

Publication [68] focused on methods for determining viscoelastic properties under conditions of low influence of surface interactions and in the presence of small deformations and compared them with switching experiments induced by turbulent flow. Another factor that strongly influences the experimental results is the anchoring of the surface. As a rule, elasticity and viscosity coefficients were measured in thin, planar, surface-stabilized samples. Surface interactions are quite weak in homeotropic cells. Using a typical ferroelectric liquid crystal, it has been shown that the correct values of the aforementioned material constants in the chiral smectic C* phase can be best determined using thick and homeotropically ordered samples. However, this method has a considerable limitation. Namely, obtaining homogeneous ordering in a thick, homeotropic sample without using a very strong electric field is extremely difficult. Determination of material constants in volumetric samples is important for material characterization, while the most common applications of ferroelectric and antiferroelectric liquid crystals are in displays, modulators, or smart films, where the material is surface-stabilized.

**Figure 5:**
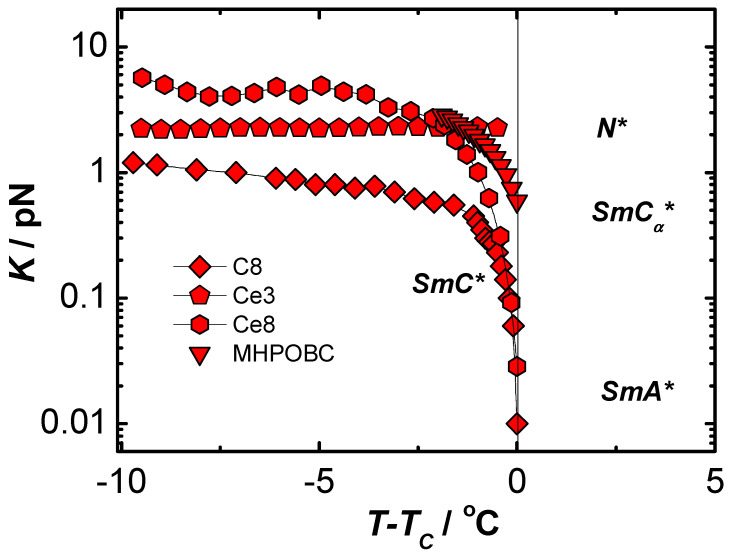
Temperature dependence of the elastic coefficient $K_{\varphi }$ in chiral liquid crystals with a ferroelectric phase: C8 (rhombs), Ce3 (diamonds), Ce8 (hexagons), and MHPOBC (triangles) obtained by electro-optical method using low electric fields.

Figure 5 collects the results obtained for various liquid crystal materials with ferroelectric properties obtained by the new electro-optical method described in [65] using weak electric fields.

**Figure 6:**
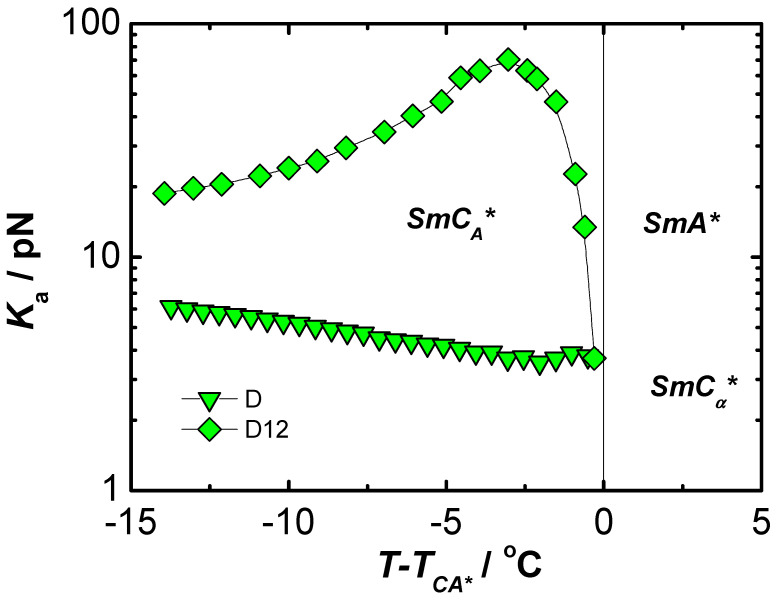
Temperature dependence of the elastic coefficient $K_{a}$ in chiral liquid crystals with antiferroelectric phase: D12 (rhomboids) and MHPOBC (triangles) obtained by electro-optical method using low electric fields.

From the results obtained, it was observed that the nature of the temperature dependence of the elastic coefficients $K_{\varphi }$ strongly depends not only on the type of liquid crystal but much more on the nature of the phase transition to the adjacent phase. The electro-optic coefficient disappears rapidly (and, in practice, radically changes its value and character) in the case of the transition to the, also chiral, cholesteric N* phase. Moving to the paraelectric A* phase, the elastic coefficient $K_{\varphi }$ begins to decrease rapidly approaching the phase transition and disappears at the temperature of the transition to the nonchiral phase. The situation is different for the antiferroelectric liquid crystal MHPOBC, in which the ferroelectric phase is bordered on both sides by chiral ferroelectric phases: SmC_α_* and SmC_γ_*. In this case, it can be assumed that $K_{\varphi }$ will vanish monotonically only in the SmC_α_* phase. Figure 6 shows the results of the temperature dependence of the coefficient of confinement Ka for chiral liquid crystals with phases with antiferroelectric properties D12 [66] and MHPOBC [71].

As shown in Figure 6, $K_{a}$ exhibits a fairly strong temperature dependence, especially near the critical temperature of the transition from the SmC* to SmA* phase. In the paraelectric phase, the parameter $K_{a}$ has a value of zero (by definition). It is also noteworthy that $K_{a}$ takes relatively large values compared to the generally accepted magnitudes of the elastic constant in ferroelectric liquid crystals (Figure 3) [68]. This can be interpreted as a stiffening of the system due to the nearly antiparallel orientation of the electric moments of molecules in adjacent smectic layers. As the temperature increases below the critical temperature $T_{C}$, p increases and δP simultaneously decreases, leading to an increase in the time-averaged dipole–dipole interaction energy between molecules of adjacent smectic layers [66]. Consequently, as the temperature increases, the stiffening effect becomes stronger and, thus, $K_{a}$ initially increases. The subsequent sharp decrease in the elastic coefficient $K_{a}$ near $T=T_{C}$ is caused by the rapid disappearance of the tilt angle in the critical region. The elasticity constant in the MHPOBC material in the antiferroelectric phase gently decreases its value with increasing temperature.

**Figure 7:**
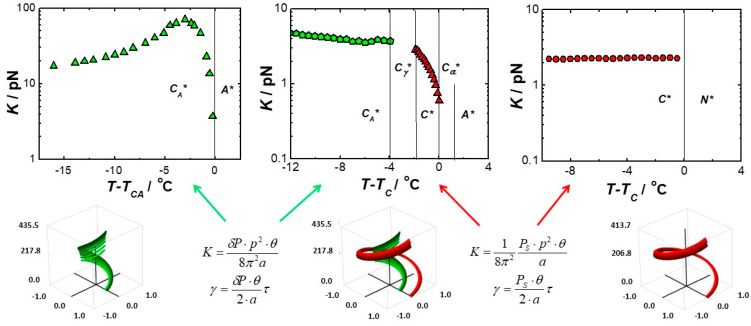
Elasticity constant $K_{\varphi }$ as a function of temperature for three different materials in the low electric field range: D12, MHPOBC, and Ce 3 showing the different dependence of the elastic constant depending on the type of adjacent phases. To illustrate the difference in helix behavior, schematic drawings are shown for one pitch period for the compounds studied. Green indicates the antiferroelectric phase C_A_*, red the ferroelectric phase C*.

The scatter of experimental results obtained for different methods and for different materials is quite large. For constant elasticity and viscosity, the experimental results can differ by orders of magnitude. The reasons for these discrepancies in helicoidal materials may be due to various aspects: on the one hand, nonlinear effects (the definitions of viscosity and elasticity coefficients assume linear torque dependence of angular velocity and strain, which is only true for laminar flows and small deformations) and, on the other hand, boundary conditions such as confining surfaces, anchorage, and gauge cell thickness (especially relevant for the direction of the screw, which, in thin planar cells, may be underdeveloped or deformed). An additional cause of discrepancies (as illustrated in Figure 7) turns out to be the influence of the sequence of phases, their chirality, and susceptibility to the electric field (the same phase in different materials shows different viscoelastic effects due to the proximity of a chiral or nonchiral, dielectric or paraelectric phase, etc.). Therefore, large deformations or turbulent flows during measurement can cause significant systematic errors. For this reason, viscoelastic properties should also be measured under defined, reproducible conditions, taking into account the helix pitch and its undisturbed distribution.

In general, the determination of viscoelastic properties is not a simple task, as previously highlighted. The main problem is the multitude of physical parameters needed to determine the values of the elasticity and viscosity constants. It is also crucial to take into account the nature of the phase in question. This issue is particularly relevant for chiral phases, such as ferroelectric and antiferroelectric phases. There are several experimental methods for measuring viscosity and elasticity constants (especially viscosity) in chiral phases. These methods use various phenomena to detect deformation, such as light transmission, polarization current, light modulation, dielectric constant, and deformation or helix unfolding. Typically, an external electric field is used to induce deformation, and it is important that the electric field is uniform within the cell. Methods for testing viscoelastic properties are relatively well defined for liquid crystalline materials with nematic phases. Over the years, this methodology has been developed and extended both theoretically and experimentally. There are now new reports on, for example, chiral active nematicides (BPs) [78] or nonchiral nematics with ferroelectric properties [79], in which it has been shown that the polar orientation order of molecules can induce chirality in soft matter without chemically induced chiral centers, which consequently also affects their rheological properties. New technological solutions are already emerging, for example INSTEC’s automatic liquid crystal testing (ALCT), which allows the automated determination of splay K_11_, bend K_33_, and twist K_22_ elastic constants in nematics with positive and negative dielectric anisotropy [80]. The situation with smectic phases is much more complicated. Quite a few theoretical models and experiments based on computer simulations are being developed. Simulations concern systems with nematic phases and smectic phases, with and without defects, with properties of hybrid systems with micro- and nanoparticles [81]. Theoretical models include assumptions to simplify calculations, for example, that smectic layers are incompressible (the angle of inclination θ remains constant), isotropies of elastic constants are assumed, or the absence of flexoelectricity and dielectric coupling. Methods for determining rotational viscosity using strong electric fields are being systematically extended, modified, and improved. There is also the possibility of automated measurement of FLC rotational viscosity proposed by INSTEC, using a specialized measurement attachment [80]. Our group focused on chiral ferroelectric and antiferroelectric liquid crystals in surface-stabilized cells. Sample homogeneity, a linear range of light modulation depth from the applied voltage, was assumed. Simultaneous calibration synchronized with the measurement of the electro-optical response provides independence from the influence of existing defects, dispersion, electric field effects, or ageing effects. To date, the selected materials have also not shown water absorption effects. In future work, it will be important to consider additional parameters that may affect the viscoelastic properties of chiral liquid crystals (temperature, humidity, homogeneity, ageing, doping, etc.). Uniformity and generalizability of conclusions is a priority for future research and solutions. The creation of a broad and accessible experimental database can be an indispensable source of information to optimize research and select the most effective methods for determining the elasticity and viscosity constants of liquid crystals [82]. Given the multitude of approaches, solutions, and questions, it is possible that machine learning will prove to be a helpful solution for the future.

## 5. Conclusions

Viscoelastic properties are one of the most fundamental properties of chiral liquid crystals. In general, their determination is not a simple task. The main problem is the multitude of physical parameters needed to determine the values of elasticity and viscosity constants. It is also necessary to take into account the nature of the phase in question. This problem is particularly important for chiral phases, such as ferroelectric and antiferroelectric phases. There are several experimental methods for measuring viscosity and elasticity constants in chiral phases. These methods use various phenomena to detect deformation, such as light transmission, polarization current, light modulation, dielectric constant, and helix deformation or unfolding. Usually, an external electric field is used to induce deformation, and it is essential that the electric field is uniform within the cell. The method of determining the coefficient of elasticity $K_{a}$ or rotational viscosity γ is often determined by the purpose for which the material under study is to be used. If we need information on mechanical constants of the volumetric type, then the use of a thick homeotropic cell for all the parameters to be determined proves to be the most reliable. Liquid crystal materials with ferroelectric and antiferroelectric properties are most often used in light modulators, displays, or smart films, where the liquid crystal is surface-stabilized. In this case, a method using a linear electro-optical coefficient a to determine viscoelastic properties while maintaining the low deformation condition is most suitable.

Nevertheless, the main challenge should be a more critical and unambiguous analysis of the observed discrepancies. Based on the results and observations, the conclusion is that further research is needed that takes into account a larger number of materials, combinations of phases, and standardized experimental conditions that will allow the generalization of the obtained conclusions.

## Figures and Tables

**Table 1:** Methods used to determine the rotational viscosity coefficient γ in chiral smectics; τ—relaxation time, $\tau_{s}$—switching time, A—electrode area, $K_{\varphi }$—torsional elasticity constant for the c-director, E—electric field, i—light intensity, P—spontaneous polarizations.

Detection Method	Voltage Shape	Equation	Literature
Light transmission (switching)	Rectangle	$\gamma =\tau_{s}PE$	[39,40]
Polarization current	Rectangle	$\gamma =0.556\tau_{s}PE$	[41]
	Triangle	$\gamma =AP^{2}E_{max}/i_{max}$	[42]
Light modulation	Harmonic	$\gamma =Kq^{2}\tau$	[43,44]
Dielectric constant	Harmonic	$\gamma =Kq^{2}\tau$	[21,29]

**Table 2:** Methods for determining the torsional elastic constant $K_{\varphi }$ in chiral smectics C*; θ—molecular tilt angle, $E_{c}$—critical field strength for the helix unfolding, q=2π/p-wave vector of the helix, p—helix pitch, ϑ—inclination of the helix axis, $\varepsilon_{0}—$electrical permittivity of the vacuum, Δε—dielectric anisotropy, P—spontaneous polarization.

Detection Method	Equation	Literature
Helix deformation Helix unwinding	$K=(16PE_{c})/(\pi^{2}q^{2})$	[45,46]
Light modulation	$K=(PE\theta )/(2\upsilon q^{2})$	[43,44]
Dielectric constant	$K=P^{2}/(2\varepsilon_{0}\Delta \varepsilon q^{2}\theta^{2})$	[47,48]

**Table 3:** Methods used to determine the elastic coefficient $K_{\varphi }$ and $K_{a}$ in chiral smectics with ferroelectric and antiferroelectric properties. The gray color indicates the new methods described in [65,66,69,71].

Detection Method	Equation	Literature
Helix deformation Helix unwinding	$K_{\varphi }=(16PE_{c})/(\pi^{2}q^{2})$	[45,46]
Light modulation	$K_{\varphi }=(PE\theta )/(2\upsilon q^{2})$	[43,44]
Dielectric constant	$K_{\varphi }=P^{2}/(2\varepsilon_{0}\Delta \varepsilon q^{2}\theta^{2})$	[47,48]
Light modulation	$K_{\varphi }=\frac{P_{S}\cdot E_{0}\cdot \theta }{2\vartheta_{0}\cdot q^{2}}$	[65]
Light modulation	$K_{a}=\frac{P_{S}\cdot E_{0}\cdot l_{0}\cdot sin2\theta }{4\vartheta_{0}\cdot q^{2}}$	[66]
Light modulation	$K_{\varphi }=\frac{1}{8\pi^{2}}\frac{P_{S}\cdot p^{2}\cdot sin\theta }{a}$	[69]
Light modulation	$K_{a}=\frac{1}{8\pi^{2}}\frac{{\delta P}_{S}\cdot p^{2}\cdot sin\theta }{a}$	[71]

**Table 4:** Methods used to determine the viscosity coefficient of γ and $\gamma_{a}$ in chiral smectics with ferroelectric and antiferroelectric properties. Gray color indicates new methods described in [62,70,71].

Detection Method	Voltage Shape	Equation	Literature
Light transmission (switching)	Rectangle	$\gamma =\tau_{s}PE$	[39,40]
Polarisation current	Rectangle	$\gamma =0.556\tau_{s}PE$	[41]
	Triangle	$\gamma =AP^{2}E_{max}/i_{max}$	[42]
Light modulation	Harmonic	$\gamma =K_{\varphi }q^{2}\tau$	[43,44]
Dielectric constant	Harmonic	$\gamma =K_{\varphi }q^{2}\tau$	[21,29]
Light modulation	Harmonic	$\gamma =\frac{P_{S}\cdot E_{0}\cdot \theta }{2\vartheta_{0}}\tau$	[63]
Light modulation	Harmonic	$\gamma =\frac{P_{S}\cdot \theta }{2a}\tau$	[70]
Light modulation	Harmonic	$\gamma_{a}=\frac{{\delta P}_{S}\cdot \theta }{2a}\tau$	[71]

**Table 5:** Liquid crystalline materials studied.

Short Name	Chemical Structure	Phase Sequence
C8	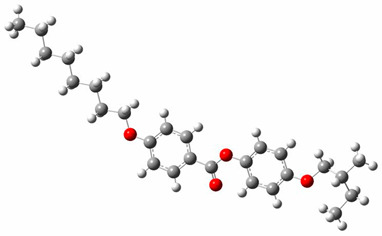	Cr 48 (SmIV 12 SmIII 21) SmC* 43.1 SmA* 59 Iso
Ce-3	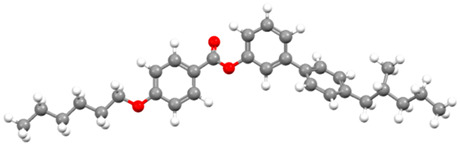	Cr 65 SmC* 77.5 N* 162 Iso
Ce-8	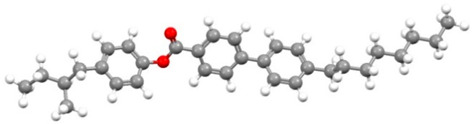	Cr 48 SmG 63.3 SmJ 64.7 SmF 66.7 SmI 69 SmC* 85 SmA* 135.4 N* 140.7 BP 141 Iso
Ce-3/8 mixture	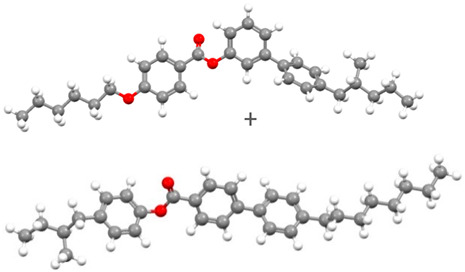	Cr 39.6 SmG 56 SmJ 65 SmF 67 SmC* 86 SmA*124 N*145.5 BP 147 Iso
D12	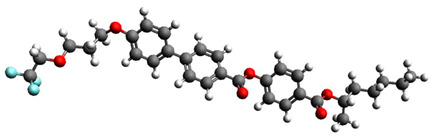	Cr 111.5 SmC_A_* 125 SmA* 136 Iso
MHPOBC	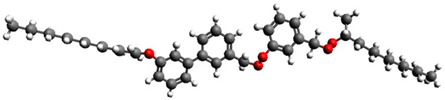	Cr 59.0 SmC*_A_ 119.5 SmC*_γ_ 120.5 SmC^*^_β_ 121.9 SmC*_α_ 123.0 SmA* 150.2 Iso

## Data Availability

The raw data supporting the conclusions of this article will be made available by the authors on request.
